# BETA-HYDROXYBUTYRATE IS A METABOLIC REGULATOR OF PROTEOSTASIS IN THE AGING BRAIN

**DOI:** 10.1093/geroni/igae098.1870

**Published:** 2024-12-31

**Authors:** Sidharth Madhavan, Stephanie Roa Diaz, Mitsunori Nomura, Christina King, Thelma Garcia, Birgit Schilling, Asish Chaudhuri, John Newman

**Affiliations:** Buck Institute for Research on Aging, Novato, California, United States; Buck Institute for Research on Aging, Novato, California, United States; Buck Institute for Research on Aging, Novato, California, United States; Buck Institute for Research on Aging, Novato, California, United States; Buck Institute for Research on Aging, Novato, California, United States; Buck Institute for Research on Aging, Novato, California, United States; Buck Institute for Research on Aging, Novato, California, United States; Buck Institute for Research on Aging, Novato, California, United States

## Abstract

Loss of proteostasis is a hallmark of aging and Alzheimer disease (AD). Here, we identify beta-hydroxybutyrate (BHB), a ketone body, as a molecular chaperone which regulates protein solubility in the brain. BHB is a small molecule metabolite which primarily provides an oxidative substrate for ATP during hypoglycemic conditions, and also regulates other cellular processes through covalent and noncovalent protein interactions. We demonstrate BHB-chaperone activity across in vitro, ex vivo, and in vivo mouse systems. This activity is shared by select structurally similar metabolites, is not dependent on covalent protein modification, pH, or solute load, and is observable in mouse brain in vivo after delivery of a ketone ester. Furthermore, this phenotype is selective for pathological proteins such as amyloid-beta (Ab), and exogenous BHB ameliorates pathology in nematode models of Ab aggregation toxicity. We have generated a comprehensive atlas of the BHB-induced protein insolublome ex vivo and in vivo using mass spectrometry proteomics, and have identified common protein domains within BHB target sequences. Finally, we show enrichment of neurodegeneration-related proteins among BHB targets and the clearance of these targets from mouse brain, likely via BHB-induced autophagy. Overall, these data indicate a new metabolically regulated mechanism of proteostasis relevant to aging and AD.

